# Spermidine Suppresses Peripheral Inflammation and Alleviates Non-Motor Symptoms in the 6-OHDA-Induced Rat Model of Parkinson’s Disease

**DOI:** 10.3390/molecules31071164

**Published:** 2026-03-31

**Authors:** Beata Grembecka, Oliwia Harackiewicz, Jan Ruciński, Daria Korewo-Labelle, Ewelina Kurowska-Rucińska, Irena Majkutewicz

**Affiliations:** 1 Department of Animal and Human Physiology, Faculty of Biology, University of Gdansk, 80-309 Gdansk, Poland; oliwia.harackiewicz@ug.edu.pl (O.H.); jan.rucinski@ug.edu.pl (J.R.); ewelina.kurowska@ug.edu.pl (E.K.-R.); irena.majkutewicz@ug.edu.pl (I.M.); 2 Department of Physiology, Faculty of Medicine, Medical University of Gdansk, 80-211 Gdansk, Poland; daria.korewo-labelle@gumed.edu.pl

**Keywords:** spermidine, Parkinson’s disease, 6-hyroxydopamine, anhedonia, anxiety, inflammation, neurodegeneration, T lymphocytes

## Abstract

Non-motor symptoms of PD impair quality of life and remain challenging to treat. Here, we examined the effects of short- (38 days) and long-term (178 days) supplementation with the natural polyamine spermidine on anhedonia and anxiety-like behaviours in a 6-hydroxydopamine-induced rat model of PD and linked them with spermidine’s anti-inflammatory properties. Behavioural assessments (cylinder, sucrose preference, elevated plus-maze tests) were conducted during progressive neurodegeneration and after oral treatment. Under the same conditions, peripheral inflammation was evaluated by the total leukocytes and their subpopulation numbers (hematological analysis) and by CD4^+^ and CD8^+^ T lymphocyte percentages (imaging flow cytometry); the plasma levels of interleukins 4 and 10 and corticosterone (enzyme-linked immunosorbent assay) were also evaluated. The safety of long-term supplementation was assessed using standard biochemical markers (chemistry analyser). Both treatment regimens reversed 6-hydroxydopamine-induced lymphopenia. Long-term spermidine treatment increased the number of TCD4^+^ lymphocytes and monocytes and elevated the plasma concentrations of IL-4 and IL-10, while reducing corticosterone levels. These immunomodulatory effects were associated with reduced anhedonia and anxiety. All of the biochemical safety parameters remained within normal ranges. Spermidine alleviates neuropsychiatric symptoms in a rat model of progressive neurodegeneration in the nigrostriatal system through its regulatory influence on peripheral immune responses. Exploring the systemic mechanisms underlying spermidine’s effects could unveil innovative supplementation strategies and expand treatment options for managing symptoms in PD.

## 1. Introduction

The non-motor symptoms of Parkinson’s disease (PD) are a significant cause of progressive deterioration in patients’ quality of life [1] and a challenge for clinicians due to the need to choose therapies primarily focused on alleviating axial symptoms [2]. Almost half (47.9%) of patients suffering from advanced PD experience depression, 25.4% have anhedonia, and 54.5% suffer from anxiety disorders and exhibit various autonomic nervous system symptoms (e.g., urinary problems, difficulty swallowing) [3]. Currently, none of the available treatment methods significantly modify the neurodegenerative processes occurring in the central nervous system (CNS), especially in the nigrostriatal system [4]. One of the reasons for therapeutic limitations is the still-unknown mechanism of dopaminergic neurons’ death [5]. The risk of developing PD is determined by environmental factors such as exposure to environmental pollutants, including pesticides [6,7], in a manner dependent on age and gender [8]. Recently, the mechanisms leading to the aggregation of misfolded alpha-synuclein (α-syn) [9] and the interactions between genetic and environmental factors leading to oxidative stress, neuro-inflammation, and altered autophagy processes have been of particular interest [10]. The proportion of the genetic form of PD in all patients is 10% [11]. Therefore, it remains crucial to consider environmental factors in research on new therapeutic strategies. One of the widely spread and well-established neurotoxic models of PD induced in rodents is the model using 6-hydroxydopamine (6-OHDA). This model recapitulates the progression of neurodegeneration in the nigrostriatal system [12] and the behavioural symptoms typical of PD [13]. The 6-OHDA-induced model of PD has an advantage over genetic and pre-fibrillar α-syn injection models, in which the complexity of the observed symptoms depends on the type of protein (human vs. rodent) or mutation [13,14,15].

One of the theories emphasizing depression in PD links low mood and anhedonia to chronic inflammation and activation of the hypothalamic–pituitary–adrenal (HPA) axis [16,17]. In advanced PD, a reduced percentage of TCD4^+^ lymphocytes in the peripheral blood has been demonstrated, which is associated with pro-inflammatory lymphocyte infiltration through the disrupted blood–brain barrier (BBB) into the CNS [18]. In the course of PD, an altered secretion profile of tumour necrosis factor alpha (TNF-α), interleukin (IL)-6, IL-1β, IL-10 and IL-4 [19,20] has been found, which may be a prognostic factor in predicting PD progression [21]. The altered cytokine secretion profile in PD co-occurs with changes in the number of each T cell subpopulation [20,22]. During inflammation, lymphocytes produce signalling substances such as cytokines, which have the ability to penetrate the CNS [23,24] and affect brain regions that control the level of HPA-axis activation and mood, such as the amygdala [25], hypothalamus [26] and prefrontal cortex [27,28].

Immunotherapies are worth mentioning among the latest therapeutic approaches in PD [29,30,31]. In particular, those that can activate natural repair mechanisms and inhibit over-activated inflammatory cascades seem to play key roles. Studies on animal models show that natural polyamines such as spermidine (SPD) are strong modifiers of ageing processes and have a potential impact on factors relevant to the pathophysiology of neurodegenerative diseases (ND) [32]. Results of animal studies [33,34] and comparative *post mortem* analyses of human brain material [35,36] indicate changes in SPD concentrations in the brain with age. Particularly sensitive regions are the basal ganglia and the frontal cortex [37], which are the brain regions affected by PD-related neurodegeneration and are associated with mood regulation. The anti-ageing properties of SPD may be due to its effect on histone acetylation [38] and protein acetylation [39] and should also be considered in an aspect not directly related to the translational apparatus, i.e., in the area of its effect on oxidative stress, autophagy and immune mechanisms. The multidirectional effects of SPD, which can occur at epigenetic, enzymatic and physiological levels, make it a substance with significant therapeutic potential. Considering its anti-inflammatory and antioxidant properties, we decided to investigate whether short- (2–38 days) or long-term (2–178 days) SPD supplementation applied during progressive neurodegeneration evoked by intra-striatal injection of 6-OHDA would prevent neurodegeneration-induced activation of the peripheral immune system. The peripheral immune system activation level was assessed based on lymphocyte count, number of the TCD4^+^ and TCD8^+^ subpopulations, and IL-4 and IL-10 cytokine concentration. At the same time, we conducted a behavioural screening to determine the severity of motor symptoms (sensorimotor performance in the cylinder test), level of anxiety (in the elevated plus-maze test; EPM) and anhedonia (sucrose preference test). Considering the impact of HPA-axis activation on the immune system and mood, we measured how long-term SPD supplementation affects plasma corticosterone (CORT) concentration. We also traced the safety profile of long-term SPD administration by examining its impact on kidney and liver function at the level of diagnostic biochemical functional indicators.

## 2. Results

### 2.1. Long-Term SPD Treatment Alleviates Motor Impairment in the 6-OHDA Rat Model of PD

**Figure 1:**
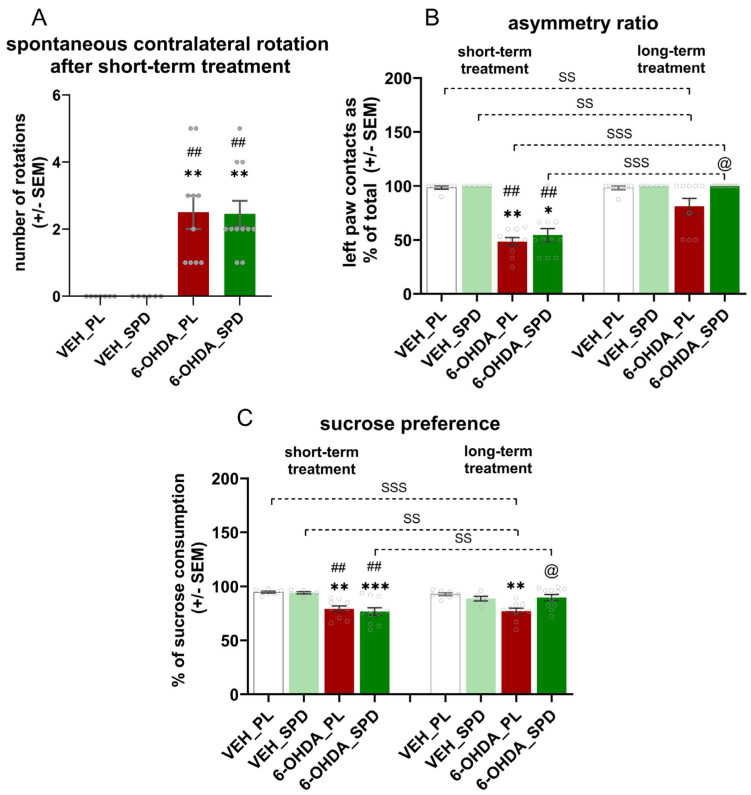
Effects of spermidine treatment on sensorimotor function and depressive-like behaviour in the 6-hydroxydopamine (6-OHDA)-injected rats during short- and long-term administration of spermidine (SPD) or placebo (PL). (**A**) Spontaneous contralateral rotations, (**B**) asymmetry ratio, and (**C**) sucrose preference ratio in the 6-OHDA-injected groups given placebo (6-OHDA_PL, *n* = 10) or spermidine (6-OHDA_SPD, *n* = 11) short- and long-term treatment, and in rats from control groups (VEH_PL, *n* = 7; VEH_SPD, *n* = 6). *** *p* < 0.001, ** *p* < 0.01, * *p* < 0.05 compared with VEH_PL for the same time point (short- or long-term supplementation); ## *p* < 0.01, compared with VEH_SPD for this same time point (short- or long-term supplementation); @ *p* < 0.05 compared with 6-OHDA_PL for the same time point (short- or long-term supplementation); SSS *p* < 0.001, SS *p* < 0.01, time-dependent differences between groups and significance according to the post hoc test are indicated in the results description (Tukey post hoc, following one-way ANOVA or post hoc Dunn following Kruskal–Wallis tests).

In both 6-OHDA-injected groups, we noted spontaneous contralateral rotations after the rat was placed in the cylinder at the first measurement point. The Kruskal–Wallis (H = 25.19; *p* < 0.0001; η^2^ = 0.74) and post hoc Dunn tests confirmed that the number of spontaneous rotations in the 6-OHDA_PL and 6-OHDA_SPD groups differed from that of the control groups (*p* < 0.01 for all comparisons) (Figure 1A) but was similar in both 6-OHDA-injected groups. As shown in Figure 1A, the contralateral forelimb (left paw) used during exploration in the cylinder test differed between groups at both points of measurement, which was confirmed by the Kruskal–Wallis test (H = 48.78; *p* < 0.001; η^2^ = 0.69). We observed sensorimotor deficits in 6-OHDA-injected animals after short-term supplementation with a placebo as compared to the VEH_PL (*p* < 0.01) and VEH_SPD (*p* < 0.01) groups. In the 6-OHDA_SPD short-term-supplemented group, the asymmetry ratio was similar to the 6-OHDA_PL group and was higher than the ones noted in the VEH_PL (*p* < 0.05) and VEH_SPD (*p* < 0.01) groups (Figure 1B). Due to a compensatory mechanism after 26 weeks of PD-model induction, the asymmetry ratio improved in both of the 6-OHDA-injected groups. However, the Dunn test confirmed that in the 6-OHDA_SPD group the asymmetry ratio was lower than in the 6-OHDA_PL group (*p* < 0.05) and was similar to that observed in both control groups (Figure 1B). In addition, there were time-dependent differences in asymmetry ratio between groups (6-OHDA_SPD_short vs. 6-OHDA_SPD_long: *p* < 0.001; 6-OHDA_PL_short vs. 6-OHDA_PL_long: *p* < 0.001).

### 2.2. Time-Dependent Effects of SPD Treatment on Depression-like Behaviour and Anxiety Level in the 6-OHDA Rat Model of PD

Anhedonic behaviour, a key feature of depressive-like behaviour, was tested with the sucrose preference test. Preference for a sweet solution was significantly affected by 6-OHDA injection in rats at both time points (F_(3,30)_ = 10.83; *p* < 0.0001; η^2^ = 0.52 and H = 13.40; *p* < 0.01; η^2^ = 0.35, respectively). The sucrose preference ratio was lower in both 6-OHDA-injected groups than in the VEH_PL (*p* < 0.01; *p* < 0.001) and VEH_SPD (*p* < 0.01; *p* < 0.01) groups at the first measurement point (Figure 1C). A low sucrose preference ratio was also observed in the 6-OHDA-injected rats after long-term placebo treatment compared to VEH_PL (*p* < 0.01). However, the long-term administration of SPD abolished the effect of nigral damage.

After long-term SPD treatment, the preference for sucrose was higher in 6-OHDA_SPD rats than in the 6-OHDA_PL (*p* < 0.05) group. Furthermore, comparative analysis (H = 35.01; *p* < 0.0001; η^2^ = 0.46) of the sucrose preference ratio between time points showed significant differences between the effects of short- and long-term treatment for the following comparisons: VEH_PL_short vs. 6-OHDA_PL_long (*p* < 0.001), VEH_SPD_short vs. 6-OHDA_PL_long (*p* < 0.01) (Figure 1C).

**Figure 2:**
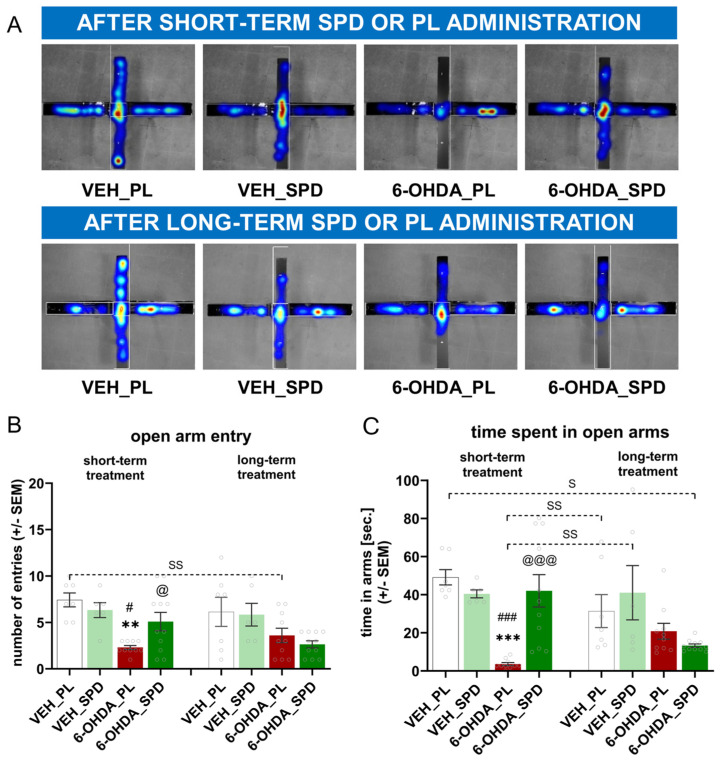
Effects of spermidine treatment on anxiety-like behaviour assessment in the 6-hydroxydopamine (6-OHDA)-injected rats during short- and long-term administration of spermidine (SPD) or placebo (PL). (**A**) Representative heatmaps from EthoVision XT10 software show elevated plus-maze (EPM) exploration in the closed (horizontal) or open (vertical) arms in representative rats for each group. Color intensity ranges from cold (blue) to hot (orange/red), reflecting the relative frequency of rat location sampling, with warmer colors indicating a higher visit frequency at a given location (**B**) number of open-arm entries and (**C**) time spent in open arms in the 6-OHDA-injected groups given placebo (6-OHDA_PL, *n* = 10) or spermidine (6-OHDA_SPD, *n* = 11) short- and long-term treatment and in rats from control groups (VEH_PL, *n* = 7; VEH_SPD, *n* = 6). *** *p* < 0.001, ** *p* < 0.01, compared with VEH_PL for the same time point (short- or long-term supplementation); ### *p* < 0.001, # *p* < 0.05 compared with VEH_SPD for this same time point (short- or long-term supplementation); @@@ *p* < 0.001, @ *p* < 0.05 compared with 6-OHDA_PL for the same time point (short- or long-term supplementation); SS *p* < 0.01, S *p* < 0.05 time-dependent differences between groups and significance according to the post hoc test are indicated in the results description (Tukey post hoc, following one-way ANOVA or post hoc Dunn following Kruskal–Wallis tests).

To evaluate anxiety-like behaviour, we measured the number of entries and time spent in open arms using the EPM test (Figure 2A). A high level of anxiety, reflected by a decrease in open-arm activity [entries (F_(7,60)_ = 4.75; *p* < 0.001; η^2^ = 0.36) and time spent in arms (H = 40.77; *p* < 0.0001; η^2^ = 0.56)], was confirmed 21 days (short-term effects) after PD-model induction in the 6-OHDA_PL group compared to VEH_PL (*p* < 0.01; *p* < 0.001) and VEH_SPD (*p* < 0.05; *p* < 0.001), as shown in Figure 2B,C. Short-term SPD administration in the 6-OHDA-injected rats elevated the number of open-arm explorations, as well as the time spent in those arms (*p* < 0.05; *p* < 0.001) compared to the 6-OHDA_PL group. The anxiety level reassessed at 25 weeks (long-term effects) was generally lower than at the first measuring point in all groups. Comparing the results of short- and long-term administration, significant differences in the time spent in open arms were noted between: the VEH_PL_short vs. 6-OHDA_SPD_long (*p* < 0.05), VEH_PL_long vs. 6-OHDA_PL_short (*p* < 0.01), and VEH_SPD_long vs. 6-OHDA_PL_short (*p* < 0.01) groups, and differences in the number of entries were observed between: VEH_PL_short vs. 6-OHDA_PL_long (*p* < 0.01).

### 2.3. Long-Term SPD Treatment Did Not Affect Nigral Degeneration in the 6-OHDA Model of PD

**Figure 3:**
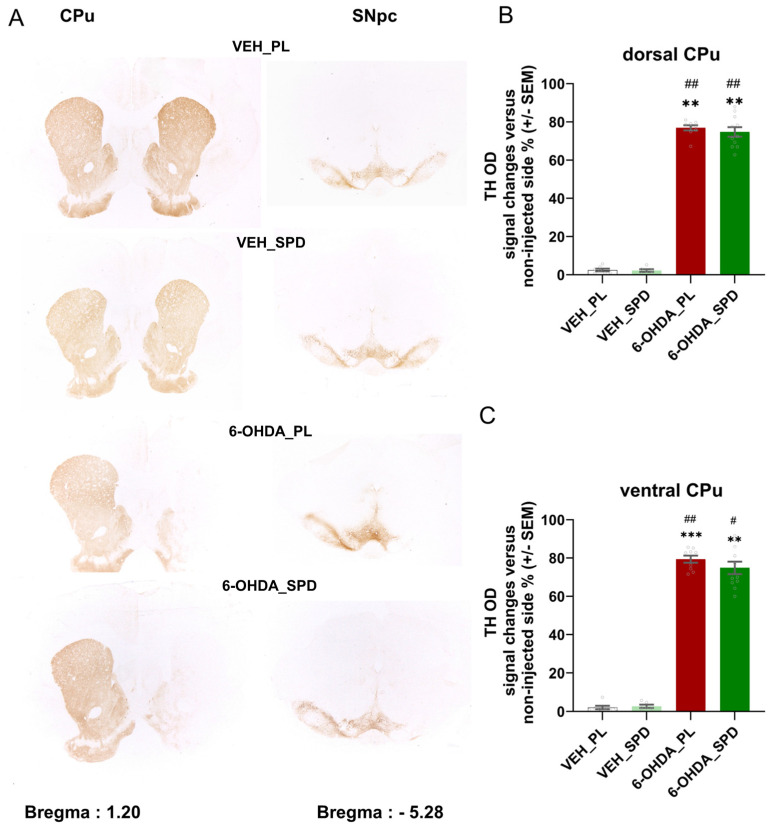
Effects of spermidine treatment on neurodegeneration in the 6-hydroxydopamine-induced rat model of Parkinson’s disease. Quantification of the dopaminergic cell loss in the substantia nigra pars compacta (SNpc) and their terminals in the caudate–putamen (CPu) after unilateral striatal 6-OHDA lesion. (**A**) The photomicrographs represent the designated anteroposterior levels of the rat brain in which tyrosine hydroxylase (TH-ir) fibre loss in the CPu or tyrosine hydroxylase (TH^+^) neurons in the SNpc were quantified on selected areas based on work by Paxinos and Watson (AP = 1.20 and AP = 5.28 mm relative to bregma, magnification 0.5 × 0.65 for CPu and 0.5 × 0.8 for SNpc). (**B**,**C**) Percentages of optical density (TH OD) change versus signal from non-injected side in dorsal and ventral CPu in the 6-OHDA-injected groups given placebo (6-OHDA_PL, *n* = 10) or spermidine (6-OHDA_SPD, *n* = 11) long-term treatment and in rats from control groups (VEH_PL, *n* = 7; VEH_SPD, *n* = 6). *** *p* < 0.001; ** *p* < 0.01 compared with VEH_PL; ## *p* < 0.01; # *p* < 0.05 compared with VEH_SPD, Tukey post hoc, following one-way ANOVA.

Analysis of TH-immunostained sections from the dorsal and ventral *caudate–putamen* (CPu) and cell bodies in the *substantia nigra pars compacta* (SNpc) confirmed that 6-OHDA induced a total tyrosine hydroxylase (TH-ir) fibre loss in the CPu and reduced the number of dopaminergic cells in the SNpc (Figure 3A). There were significant differences in the optical density (TH OD) across groups (H = 23.26; *p* < 0.0001; η^2^ = 0.67 for dorsal CPu and H = 23.56; *p* < 0.000; η^2^ = 0.68 for ventral CPu), but only between the 6-OHDA-injected groups and the controls (6-OHDA_PL vs. VEH_PL: *p* < 0.01 and *p* < 0.001; 6-OHDA_PL vs. VEH_SPD: *p* < 0.01 and *p* < 0.01; 6-OHDA_SPD vs. VEH_PL: *p* < 0.01 and *p* < 0.01; 6-OHDA_SPD vs. VEH_SPD: *p* < 0.001 and *p* < 0.5), as shown in Figure 3B,C. Long-term SPD administration did not impact TH-labelled cells in the 6-OHDA rat model of PD.

The level of dopaminergic denervation in the dorsal CPu reached 76.8% in the 6-OHDA_PL group and 74.76% in the 6-OHDA_SPD group. Ventral striatum denervation was 79.39% in the 6-OHDA_PL and 78.84% in the 6-OHDA_SPD groups.

### 2.4. SPD Treatment Attenuates Peripheral Inflammation in the 6-OHDA Model of PD

To link rats’ behavioural changes to the level of peripheral immune response, we examined the numbers and percentages of leukocytes, lymphocytes, monocytes, and granulocytes in peripheral blood. In addition, flow cytometric immunophenotypic analysis was used to evaluate the T lymphocyte population: TCD4+ and TCD8+.

**Figure 4:**
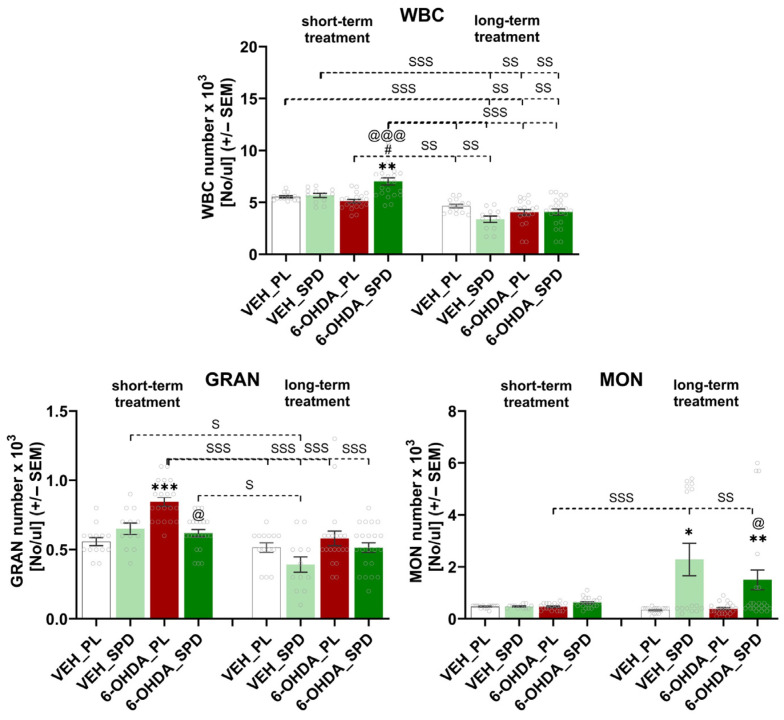
Effects of spermidine treatment on the number of each leukocyte population. Total white blood cells (WBCs) and granulocyte (GRAN) and monocyte (MON) numbers in the 6-hydroxydopamine (6-OHDA)-injected rats during short- and long-term administration of spermidine (SPD) or placebo (PL) (6-OHDA_PL, *n* = 10; 6-OHDA_SPD, *n* = 11) and in rats from control groups (VEH_PL, *n* = 7; VEH_SPD, *n* = 6). *** *p* < 0.001, ** *p* < 0.01, * *p* < 0.05 compared with VEH_PL for the same time point (short- or long-term supplementation); # *p* < 0.05 compared with VEH_SPD for the same time point (short- or long-term supplementation); @@@ *p* < 0.001, @ *p* < 0.05 compared with 6-OHDA_PL for the same time point (short- or long-term supplementation); SSS *p* < 0.001, SS *p* < 0.01, S *p* < 0.05 time-dependent differences between groups and significance according to the post hoc test are indicated in the results description (Tukey post hoc, following one-way ANOVA or post hoc Dunn following Kruskal–Wallis tests).

**Figure 5:**
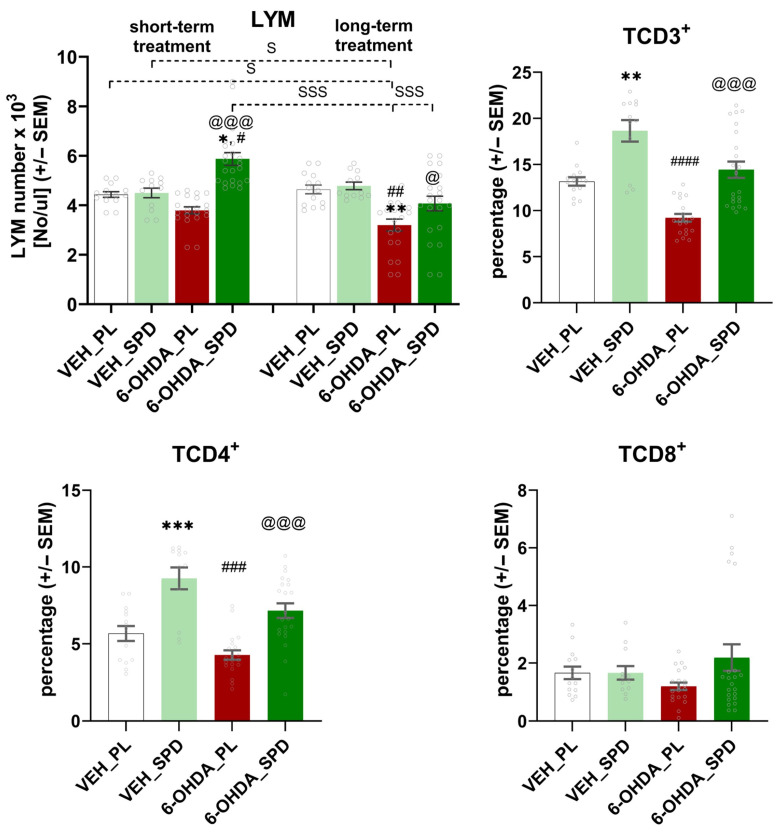
Effects of spermidine treatment on the lymphocyte population. Lymphocyte (LYM) numbers and TCD3+, TCD4+, TCD8+ percentages in the 6-hydroxydopamine (6-OHDA)-injected rats during short- and long-term administration of spermidine (SPD) or placebo (PL) (6-OHDA_PL, *n* = 10; 6-OHDA_SPD, *n* = 11) and in rats from control groups (VEH_PL, *n* = 7; VEH_SPD, *n* = 6). *** *p* < 0.001, ** *p* < 0.01, * *p* < 0.05 compared with VEH_PL for this same time point (short- or long-term supplementation); #### *p* < 0.0001, ### *p* < 0.001, ## *p* < 0.01, # *p* < 0.05 compared with VEH_SPD for the same time point (short- or long-term supplementation); @@@ *p* < 0.001, @ *p* < 0.05 compared with 6-OHDA_PL for the same time point (short- or long-term supplementation); SSS *p* < 0.001, S *p* < 0.05 time-dependent differences between groups and significance according to the post hoc test are indicated in the results description (Tukey post hoc, following one-way ANOVA or post hoc Dunn following Kruskal–Wallis tests).

The most pronounced effects on changes in the number of each leukocyte population were observed in the LYMs and GRANs (Figure 4 and Figure 5). The number of LYMs after short- and long-term SPD treatment was significantly higher (F_(7,128)_ = 13.94; *p* < 0.001; η^2^ = 0.43) in the 6-OHDA-injected rats compared with the placebo group (6-OHDA_PL: *p* < 0.001 and *p* < 0.05). Furthermore, 25 weeks after PD-model induction, the LYM number was elevated in the 6-OHDA_SPD group compared to the 6-OHDA_PL group (*p* < 0.05). We also observed lymphopenia in 6-OHDA-injected animals without SPD supplementation when compared with control groups at the same time point (VEH_PL: *p* < 0.01 and VEH_SPD: *p* < 0.01). Time-dependent effects were noted within the 6-OHDA_SPD group (long- vs. short-term treatment, *p* < 0.001), between the 6-OHDA_PL_long and controls at the first time point (VEH_PL_short: *p* < 0.05; VEH_SPD_short: *p* < 0.05) and between the 6-OHDA_SPD_short supplemented group and the 6-OHDA_PL_long supplemented group (*p* < 0.001). While the number of LYMs increased after SPD supplementation in the rat model of PD, the opposite effect was observed in the number of GRANs, especially after a short-term administration period (Figure 4). We noted that SPD treatment caused a significant reduction in GRAN number (H = 52.07; *p* < 0.0001; η^2^ = 0.35) after short-term supplementation. An elevated number of GRANs was observed in the 6-OHDA_PL group as compared with the control (VEH_PL: *p* < 0.001) and 6-OHDA_SPD groups (*p* < 0.05).

The time-dependent effect was confirmed in the 6-OHDA-injected group without SPD administration (6-OHDA_PL). In this group of rats, the GRAN number decreased over time and was reduced at the end of the procedure (*p* < 0.001) compared to the 6-OHDA_PL groups at the first time point. In addition, time-dependent changes were also noted when comparing the GRAN number in the 6-OHDA_PL at the first time point after PD-model induction with the numbers observed at the long-term period in both control groups (VEH_PL: *p* < 0.001; VEH_SPD: *p* < 0.001). As for the monocytes indices, a statistically significant (H = 35.59; *p* < 0.0001; η^2^ = 0.22) increase was noted in both SPD treatment groups when compared with the VEH_PL group, but only after long-term treatment (Figure 4). The MON number did not change significantly during PD-model progression, though we noted differences between the 6-OHDA_PL group at the first point of measuring and the VEH_SPD (*p* < 0.001) and 6-OHDA_SPD (*p* < 0.01) groups after long-term treatment (Figure 4).

The changes in the numbers of LYMs, GRANs and MONs discussed above were reflected in the significant changes (F_(7,128)_ = 19.50; *p* < 0.0001; η^2^ = 0.52) in the total number of WBCs (Figure 4). We observed that WBC number was elevated in the 6-OHDA_SPD group after short-term SPD supplementation in comparison to the VEH_PL (*p* < 0.01), VEH_SPD (*p* < 0.05) and 6-OHDA_PL (*p* < 0.001) groups (Figure 4). Furthermore, the WBC number noted in the 6-OHDA_SPD rats was higher than the values observed in all groups at the end of the procedure (VEH_PL, VEH_SPD, 6-OHDA_PL, 6-OHDA_SPD, *p* < 0.001). In addition, the WBC number in the 6-OHDA_PL rats at the first point of measurement was higher than in both control groups at the end of the procedure (VEH_PL: *p* < 0.01, VEH_SPD: *p* < 0.01, respectively). We noted a significant impact of time of supplementation on WBC number across all groups, since WBC numbers decreased during the time of the procedure. This effect was reflected by observed differences between VEH_PL at the first point of measurement and the VEH_SPD (*p* < 0.001), 6-OHDA_PL (*p* < 0.01), and 6-OHDA_SPD (*p* < 0.01) groups at the end of the procedure. The same pattern of changes was noted when comparing the VEH_SPD group after short-term supplementation with data obtained after long-term treatment in the VEH_SPD (*p* < 0.001), 6-OHDA_SPD (*p* < 0.01) and 6-OHDA_PL (*p* < 0.01) groups.

The changes in the number of leukocytes were reflected in the population percentages (%) according to the LYM (H = 33.95, *p* < 0.0001; η^2^ = 0.48) and MON (H = 12.80, *p* < 0.01; η^2^ = 0.09) values in 6-OHDA-injected rats after short-term SPD treatment, and the LYMs (H = 34.05, *p* < 0.0001; η^2^ = 0.48) after long-term SPD treatment, as shown in Table 1. A reduced LYM% was noted in the 6-OHDA_PL group compared with VEH_PL at the first (*p* < 0.05) and second (*p* < 0.01) point of measurement. SPD treatment abolishes these changes. The LYM% in the 6-OHDA_SPD group at the first point of measurement was higher than in the VEH_SPD group (*p* < 0.05) and the 6-OHDA_PL group (*p* < 0.0001). A similar effect was noted after long-term SPD treatment when comparing the 6-OHDA_SPD with 6-OHDA_PL (*p* < 0.05). An elevated MON% was observed in the 6-OHDA_SPD group after short-term SPD treatment when compared to VEH_PL (*p* < 0.05) and 6-OHDA_PL (*p* < 0.05).

### 2.5. Long-Term SPD Treatment Influence on Peripheral TCD3+ and TCD4+ Lymphocyte Percentage

As a result of chronic inflammation occurring in PD, activated pro-inflammatory T lymphocytes are redirected to the CNS and hence their number in peripheral blood is reduced [23,24]. We observed that the number of lymphocytes increased due to long-term SPD supplementation. Therefore, we performed lymphocyte immunophenotyping using flow cytometry to investigate which lymphocyte subpopulation is responsible for the observed effect. As shown in Figure 5, the percentage of TCD3+ and TCD4+ lymphocytes significantly (F_(3,64)_ = 23.47, *p* < 0.001; η^2^ = 0.52 and F_(3,64)_ = 17.96, *p* < 0.001; η^2^ = 0.42) increased in 6-OHDA_SPD rats in comparison with the 6-OHDA_PL (*p* < 0.001 and *p* < 0.001) group. The immunostimulant effects of spermidine were also observed in the VEH_SPD group, in which the counts of TCD3+ and TCD4+ were higher than in the VEH_PL (*p* < 0.01 and *p* < 0.001) group.

### 2.6. Long-Term SPD Treatment Activates Anti-Inflammatory IL-10 and IL-4 Secretion and Changes in Peripheral Blood CORT Concentration

**Figure 6:**
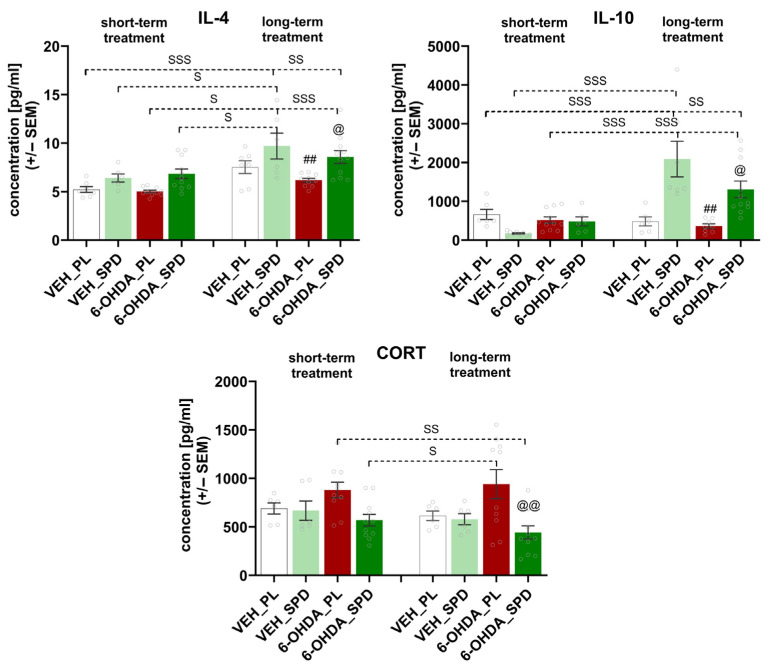
Effects of spermidine treatment on anti-inflammatory cytokines and corticosterone concentration. Interleukin (IL)-4, IL-10 and corticosterone (CORT) plasma concentration in the 6-hydroxydopamine (6-OHDA)-injected rats during short- and long-term administration of spermidine (SPD) or placebo (PL) (6-OHDA_PL, *n* = 10; 6-OHDA_SPD, *n* = 11) and in rats from control groups (VEH_PL, *n* = 7; VEH_SPD, *n* = 6). ## *p* < 0.01, compared with VEH_SPD for the same time point (short- or long-term supplementation); @@ *p* < 0.01, @ *p* < 0.05 compared with 6-OHDA_PL for the same time point (short- or long-term supplementation); SSS *p* < 0.001, SS *p* < 0.01, S *p* < 0.05 time-dependent differences between groups and significance according to the post hoc test are indicated in the results description (Tukey post hoc, following one-way ANOVA or post hoc Dunn following Kruskal–Wallis tests).

Changes in the number of TCD4^+^ lymphocytes and monocytes led to increased secretion of IL-10 and IL-4. As shown in Figure 6, significant statistical differences among the groups were demonstrated in their plasma IL-10 and IL-4 concentrations (H = 46.93, *p* < 0.0001; η^2^ = 0.71 and F_(7,60)_ = 7.626, *p* < 0.0001; η^2^ = 0.47). Prolonged administration of SPD resulted in elevated concentrations of both cytokines in peripheral blood compared with the 6-OHDA_PL groups at the first (*p* < 0.001 and *p* < 0.001) and last time points of the procedure (*p* < 0.05 and *p* < 0.05). In addition, the highest IL-10 and IL-4 concentrations were noted in the VEH_SPD groups after long-term treatment when compared with the results observed in the VEH_PL (*p* < 0.001 and *p* < 0.001) and VEH_SPD (*p* < 0.001 and *p* < 0.05) groups after short-term treatment. The anti-inflammatory effects observed in the PD-model groups were associated with CORT levels in the peripheral blood (F_(7,60)_ = 3.918, *p* < 0.01; η^2^ = 0.32). Long-term SPD treatment led to a reduction in CORT level in the 6-OHDA_SPD group compared to the 6-OHDA_PL group at both time points (*p* < 0.01 and *p* < 0.01, respectively) (Figure 6). At the first time point of CORT measuring, the results indicated a consistent trend in the direction of change, although the differences between groups were not statistically significant

### 2.7. Safety of Long-Term SPD Treatment

Due to the prolonged use of the SPD, we also evaluated its potential effects on organ function, including the kidneys, liver, and pancreas, by analyzing biochemical markers in peripheral blood (Table 2). Although the measured parameters remained within the physiological reference ranges, notable intergroup differences were observed for TB (H = 16.08, *p* < 0.01; η^2^ = 0.87), AMY (H = 12.12, *p* < 0.01; η^2^ = 0.57) and UREA (H = 8.542, *p* < 0.05; η^2^ = 0.35). The long-term SPD treatment in 6-OHDA-injected rats resulted in elevated blood AMY and UREA concentrations compared with the 6-OHDA_PL (*p* < 0.05) and VEH_SPD (*p* < 0.05) groups, respectively. A higher TB level was noted in the 6-OHDA-injected rats. Long-term SPD treatment normalized this level.

## 3. Discussion

In this study, we analyzed the short- and long-term effects of SPD treatment in the 6-OHDA-induced rat model of PD on sensorimotor, depressive-like, and anxiety-like behaviour. We linked behavioural changes with peripheral immune system activation and plasma CORT concentration. We showed that the administration of 6-OHDA into the striatum results in denervation of the CPu. The dopaminergic denervation reached 75–79% of fibres in the CPu and total loss of the cell bodies in the SNpc was noted. Along with the loss of dopaminergic neurons, we confirmed the presence of sensorimotor deficits in the cylinder test in a 6-OHDA-induced model of PD. After the lesion, rats exhibited an increased asymmetry ratio and spontaneous rotations at the first assessment point. The limb motor dysfunction observed in rats with a PD model is an expected effect of damage to the nigrostriatal system, due to 6-OHDA being administered into the striatum [40,41,42]. SPD supplementation, initiated 24 h after 6-OHDA injection, did not prevent or effectively limit the neurotoxin-induced loss of dopaminergic neurons in PD rats. Due to the lack of a neuroprotective effect within a short window of SPD administration, no changes in motor deficits were observed in the SPD-supplemented rats. Motor impairments improved at the second (late) assessment point, which is associated with compensatory mechanisms occurring in cases of unilateral lesions in the nigrostriatal system [43]. Interestingly, the compensatory effect was more pronounced in the group receiving long-term SPD supplementation. This may indicate that prolonged SPD administration in rats with a 6-OHDA-induced PD model protects against age-related motor deficits. Other animal studies highlight the impact of SPD on promoting skeletal muscle regeneration [44] and preventing age-related muscle atrophy in rodents [45]. The most recent study by Zhang and colleagues proved the mechanism of SPD influence on the skeletal muscle functioning [46] and the ability of SPD to promote muscle regeneration and delay muscle ageing [44]. Unfortunately, our research did not establish the assessment of SPD treatment on skeletal muscle physiology, therefore this aspect seems to be an interesting topic for future research.

Interestingly, we report that long-term SPD supplementation, applied 24 h after the 6-OHDA injection, did not affect the neurotoxin-induced death of dopaminergic neurons in rats. Although the neuroprotective effect of SPD was observed in the study by Sharma and colleagues [47], we did not confirm it in our research. In the rotenone-induced PD model, SPD administered orally for 14 consecutive days at a dosage of 10 mg/kg led to neuroprotection and reduced motor impairment in the rat model of PD [47]. Based on the results of previously published studies, we assume that SPD’s lack of protective effect in the 6-OHDA model is most likely due to the different mechanisms of neuronal death induced by rotenone and 6-OHDA. Rotenone is a highly lipophilic and hydrophobic compound, allowing it to readily cross cell membranes without needing specific transporters [48]. 6-OHDA requires active transport into cells via particular membrane transporters, such as the dopamine and norepinephrine transporters [49]. Furthermore, 6-OHDA is sensitive to oxidation outside the cell and it readily forms semiquinone radicals and participates in redox cycling, contributing to the generation of various reactive oxygen species (ROS), including hydrogen peroxide (H_2_O_2_), superoxide (O_2_•^−^), and hydroxyl radicals (•OH) [50]. The 6-OHDA and rotenone also differ in how they induce mitochondrial toxicity. Rotenone is a gold-standard mitochondrial complex I (NADH ubiquinone reductase) inhibitor, known for its high affinity and time-dependent and irreversible binding [51]. The 6-OHDA mechanism of action includes inhibition of I and IV (cytochrome-c oxidase) mitochondrial complexes [52]. Additionally, an in vitro study showed that 6-OHDA-induced neurotoxicity leads to mitochondrial dysfunction with a loss of MMP, a critical event in dopaminergic neuron degeneration [53]. It seems that the SPD influence on oxidative stress in the CNS [54,55,56,57] is well documented. However, there is a gap that requires defining the precise mechanism of the antioxidant properties of SPD in brain tissue. The absence of a protective effect from SPD in the 6-OHDA model may be due to the timing and dosage of its administration. In the case of rotenone, neuronal death occurs more gradually after injection, providing a longer window for intervention. In contrast, 6-OHDA acts immediately when administered in the striatum [58,59]; therefore, applying SPD 24 h later may have been too late to counteract the neurodegeneration of dopaminergic cells.

Here, we report that SPD influences sucrose preference, exploration, and time spent in the open arms of an EPM in the 6-OHDA-induced model of PD in a time-dependent manner by a mechanism involving the suppression of chronic peripheral inflammation. These interesting results emphasize that anhedonia observed in the rat model of PD depends not only on dopaminergic denervation (for review see: [60]), but rather on inflammatory activation. A growing body of evidence suggests that in the rat 6-OHDA-induced model of PD, dopamine is involved in motivational behaviour [61,62], rewarding, and hedonic processes [63,64]. In this study, we showed that anhedonia was elevated in 6-OHDA-injected rats during progressive neurodegeneration, and SPD treatment abolished these depressive symptoms after long-term treatment. Since SPD did not exert a neuroprotective effect on dopaminergic neurons in our treatment model, the mood enhancement was not only dependent on dopamine secretion. Therefore, we propose that this effect was related to SPD’s influence on the chronic peripheral inflammatory response triggered by ongoing neurodegeneration in the nigrostriatal system. Studies have demonstrated that administration of 6-OHDA as a model of PD induces a chronic inflammatory state, characterized by the pro-inflammatory activation of microglial cells [59,65,66,67,68] and peripheral lymphocyte distribution changes due to BBB perturbation [69,70,71]. In this experiment, we observed peripheral leukopenia and lymphopenia in a rat 6-OHDA-injected model of PD at both measuring points. Changes in lymphocyte number and percentage in 6-OHDA-injected rats were associated with T lymphocyte infiltration at the site of neurodegeneration, as was shown by Jiang et al. [71] and Ambrosi et al. [72]. We proved that SPD treatment restores the number of lymphocytes and the percentage of TCD4^+^ cells in the peripheral blood. We found that the retention of lymphocytes in the systemic circulation is linked to a mechanism that depends on SPD influence on monocytes and TCD4^+^ lymphocytes to promote the secretion of anti-inflammatory cytokines. Indeed, in our studies, we demonstrated that SPD induced an increase in the number of monocytes in the peripheral blood and elevated concentrations of IL-4 and IL-10, which suppressed the inflammatory response. This anti-inflammatory effect was also associated with a reduced CORT level following SPD administration.

Increasing evidence supports peripheral inflammation’s deleterious role in PD neurodegeneration (for review see: [20]). The animal studies underlined the role of peripheral TCD4^+^ lymphocytes in the pathogenesis and progression of PD [73,74]. In this study, we showed the influence of SPD treatment on the TCD4^+^ lymphocyte count in the peripheral blood of rats with the 6-OHDA-induced model of PD. Excellent research by Puleston et al. [75] confirms the results we received. In their study, loss of polyamine synthesis leads to profound changes in the ability of CD4^+^ T cells to differentiate into distinct T helper (Th) subsets. The SPD acts as a substrate for synthesizing the amino acid hypusine and mice with a T cell-specific deletion (Dohh-DT) exhibit T cell dysregulation, peripheral inflammation, and colitis. Carriche et al. [76] showed that SPD modulates CD4^+^ T cell differentiation in vitro, preferentially committing naive T cells to a regulatory phenotype. After SPD treatment, activated T cells lacking the autophagy gene Atg5 fail to upregulate forkhead box protein 3 (Foxp3) to the same extent as wild-type cells. In addition, dietary supplementation with SPD in mice promotes homeostatic differentiation of regulatory T cells (Tregs) within the gut and reduces pathology in a model of T cell transfer-induced colitis. CD4^+^CD25^+^Foxp3^+^ Tregs are immunoregulatory cells that express the master transcription factor Foxp3 and account for only 3–10% of peripheral CD4^+^ T cells [77]. Treg cells are crucial for maintaining immune tolerance by suppressing the activation, proliferation, and function of effector immune cells. They secrete anti-inflammatory cytokines such as IL-10, IL-35, and transforming growth factor beta (TGF-β) to inhibit immune cells in a contact-independent manner [78,79]. Indeed, in our study, we observed elevated plasma IL-10 and IL-4 concentrations after short- and long-term SPD treatment in 6-OHDA-injected rats. Another function of Tregs is their ability to regulate monocyte differentiation toward alternatively activated monocytes/macrophages (AAM). AAMs are cells with strong anti-inflammatory potential involved in immune regulation and tissue remodelling [80]. The influence of Tregs on monocytes led to a reduced production of pro-inflammatory cytokines (IL-6 and TNF-α) and activation of the nuclear factor kappa-light-chain-enhancer of the activated B cell (NF-κB) signalling pathway [81]. IL-6 and TNF-α levels are elevated in rats with a 6-OHDA-induced model of PD, as was shown by Gasparotto et al. [82] and Tiefensee Ribeiro et al. [83]. Furthermore, monocytes co-cultured with Tregs downregulated the expression of co-stimulatory and major histocompatibility complex (MHC)-class II molecules with a concomitant upregulation of M2 macrophage-specific markers, CD206, heme oxygenase-1, and increased IL-10 production [84]. There is a growing body of evidence that SPD promotes the anti-inflammatory properties of macrophages in the mouse experimental model of autoimmune encephalomyelitis (EAE) [84] and in dextran sulfate sodium (DSS)-induced inflammatory bowel disease (IBD) in mice [85]. In the last study by Niechcial et al. [86], SPD supplementation in Rag2-/-mice reduces intestinal inflammation by promoting anti-inflammatory macrophages, maintaining a healthy microbiome and preserving epithelial barrier integrity. Li et al. [87] showed that SPD treatment against *Staphylococcus aureus* (*S. aureus*—MRSA)-induced bloodstream infection in mice reduced the bacterial load and expression of inflammatory factors by shifting the macrophage phenotype to an anti-inflammatory phenotype, ultimately prolonging the survival of the infected mice. In our study, we observed an elevated ratio of monocytes in the peripheral blood after SPD treatment. Many studies have shown that SPD inhibits the production of pro-inflammatory cytokines such as TNF-α, IL-1β, and IL-6, which are released by activated microglia and astrocytes [88] and by peripheral macrophages [84]. Intraperitoneal injection of SPD (2 and 50 mg/kg) in collagen-induced arthritis mice inhibits macrophage polarization into an M1 pro-inflammatory phenotype in the synovial tissue, suppresses the levels of IL-6 and IL-1β in the serum, and increases the level of anti-inflammatory IL-10 [89]. In ACLT (anterior cruciate ligament transection) surgery, treatment with spermidine (at doses of 0.3, 3, and 6 mM) also reduced elevated levels of pro-inflammatory cytokines (TNF-α, IL-6, and IL-8) in the serum [90]. The changes in monocyte numbers observed in our study are likely associated with a monocyte anti-inflammatory activation triggered by SPD, as evidenced by the elevated secretion levels of IL-4 and IL-10. The impact of SPD on monocyte/macrophage function is worth noting, since Jin et al. [91] showed that exosomes from stimulated macrophages promote pro-inflammatory cytokine expression (IL-1α, IL-1β, IL-2, IL-6, IL-12β, and TNF-α) in the primary microglia and astrocytes and trigger neurodegeneration in the nigrostriatal system in mice.

Herein, we observed a reduction in anhedonia in rats following SPD supplementation. This may be attributed to the shift in monocyte/macrophage cytokine secretion patterns and the decrease in CORT level noted in our study. This finding aligns with existing research linking peripheral inflammation with mood disturbances, such as the “macrophage theory of depression” [92]. According to this theory, pro-inflammatory cytokines released by peripheral immune cells can exacerbate mood symptoms by influencing the HPA axis and stimulating CORT secretion [93,94,95,96,97]. Although we did not directly assess pro-inflammatory cytokine levels, our results showed a decrease in CORT and an increase in anti-inflammatory cytokine concentration following SPD supplementation. These changes suggest a potential shift in monocyte/macrophage activity towards an anti-inflammatory phenotype, which may underlie the observed improvement in affective behaviour after prolonged SPD supplementation. It has been reported that intranasal administration of IL-4 in mice with depressive-like behaviour ameliorated neuropsychiatric symptoms, reduced the plasma levels of CORT, restored the expression of nuclear factor erythroid 2-related factor 2 (NRF2), NF-κB, IL-1β, IL-4, brain-derived neurotrophic factor (BDNF), and indoleamine 2,3-dioxygenase (IDO) in the prefrontal cortex and hippocampus, and modulated oxidative stress markers in these brain structures in stressed mice [98].

Besides decreased anhedonia level, we noted anti-anxiety effects of SPD administration in 6-OHDA-injected animals. It is worth noting that long-term SPD treatment of middle-aged rats reduced anxiety, as indicated by an increase in the proportion of time spent in the open arms of the EPM and by an improvement in exploratory performance in the cylinder test [99]. SPD acts as a ligand of N-methyl D-aspartate acid (NMDA) receptors in a dose- [100] and time-dependent manner [101]. In ultra-low doses (0.02–2 nmol), SPD acts as a positive allosteric modulator of NMDA receptors [102], while in high doses (10 mg) it reduces glutaminergic-induced excitotoxicity, acting as an antagonist of NMDA [103,104]. Taken together, the anxiolytic-like effects of SPD treatment may be related to CORT release and influence on glutaminergic transmission in the CNS.

In this study, we analyzed the safety profile of long-term SPD treatment on liver, kidney, and pancreas biochemical markers and found that SPD treatment influences hepatic function. We showed that SPD administration in the 6-OHDA-induced model of PD decreased TB levels in the blood. Studies by Jin et al. [105] and Macías-García et al. [106] demonstrated elevated TB levels in patients with PD. In contrast, in the present study, SPD administration reduced TB concentration, suggesting a potential hepatoprotective and therapeutic effect of SPD. The absence of significant alterations in other hepatic metabolic biomarkers (AST, ALP, and ALT) further supports findings by Adhikari et al. [107] and Campreciós et al. [108], who also reported liver-protective properties of SPD. Jin et al. [109] indicated that polyamine catabolism may contribute to the early stages of pancreatic inflammation, which could explain the increase in the AMY observed in the present study. This elevation may reflect similar underlying mechanisms of SPD action. Moreover, research by Li et al. [110] identified a link between urea transporter B (UT-B) overexpression and polyamine metabolism, which is consistent with the increased UREA concentration observed in this study. However, the observed changes in parameter concentrations remained within the upper reference limits, confirming that SPD is well-tolerated and does not exert harmful effects on major organs. This supports the safety profile of SPD in prolonged use applied in the rat model of PD.

The main limitation of our study is the lack of results concerning neuro-inflammation and oxidative stress parameters in the brain and peripheral blood. In this study, we did not confirm the presence of lymphocytes in the brain and microglia/macrophage activation. However, the results from previous studies conducted by other research groups, as discussed above, provided a rationale for hypothesizing that administration of 6-OHDA leads to the activation of central inflammatory processes. Another limitation of our study is that the 6-OHDA rat model of PD does not recapitulate the accumulation of α-syn aggregates in the CNS. However, recent work by Cui et al. [111] suggests that our findings may still hold translational relevance. Specifically, their research demonstrated that α-syn deposition occurs in the colon after striatal 6-OHDA injection. Considering the dual-hit hypothesis of PD progression [112] and the well-established impact of SPD on gut microbiota eubiosis [113,114], our results may contribute to a deeper understanding of the mechanisms by which SPD exerts its effects in the rat model of PD. The other limitations included the lack of quantification of SPD or its metabolites in the plasma, brain and other organs before and after treatment. From already existing and conducted experiments in this area, it is known that orally administered SPD is able to cross the BBB, since deuterium-labelled-SPD was detectable in brain regions (hippocampus, cortex, striatum, mid brain, bulbs, and cerebellum) of C57BL/6J mice after a 1-week period of supplementation [115]. SPD can also be absorbed into the bloodstream from the gastrointestinal tract through diffusion, which is a key mechanism for rapid SPD absorption during oral administration [116,117,118]. Blankenship and Marchant [119] examined the metabolism of N1-acetylspermidine and N8-acetylspermidine, two products of spermidine acetylation, in the liver and kidneys of rats. Both SPD and spermine can be detected in, e.g., livers, kidneys, and spleens [119,120], and SPD can also be found in the urine of rats [121], proving its metabolism by mammals.

## 4. Materials and Methods

### 4.1. Animals

**Figure 7:**
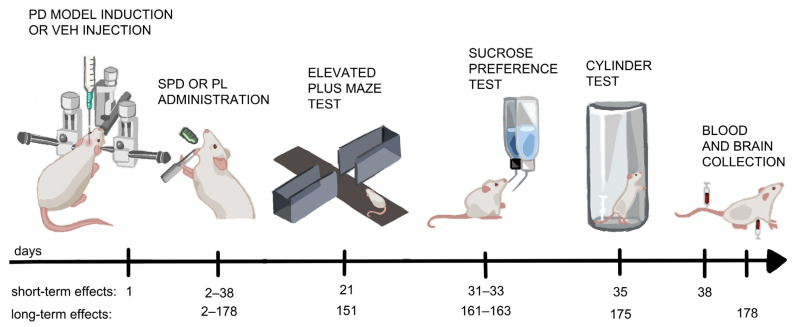
Schematic diagram of experimental timeline. Explanations: PD model—Parkinson’s disease model with 6-hydroxydopamine injection; VEH—control group with vehicle injection; short-term effects—1–38 days after PD-model induction; long-term effects—1–178 days after PD-model induction; SPD—spermidine; PL—placebo, VEH_PL (*n* = 7), VEH_SPD (*n* = 6), 6-OHDA_PL (*n* = 10), 6-OHDA_SPD (*n* = 11).

Wistar Han male rats (*n* = 34) were purchased from the Tri-City Central Animal Laboratory, Research and Service Centre of the Medical University of Gdansk (breeder registration number 041) at an age of 8 weeks and housed in cages of five until PD-model induction. Standard plastic cages with elevated metal wire lids were used, and rats were maintained on a 12:12 h light/dark cycle (lights on at 06.00 AM), with *ad libitum* access to a standard rat diet (Labofeed B standard, Morawski, Kcynia, Poland) and water. After 2 weeks of acclimatization, the animals were handled daily and adapted for the oral (*per os*, p.o.) administration procedure to minimize stress caused during experimental procedures. The handling procedure was conducted repeatedly over a two-week period. Then, the rats were randomly allocated to one of 4 groups: (1) a control group for PD-model induction (*vehiculum*, VEH) and placebo (PL) p.o. treatment (VEH_PL; *n* = 7), (2) a control group for PD-model induction and SPD p.o. treatment (VEH_SPD; *n* = 6), (3) a group with a 6-OHDA-induced model of PD and PL p.o treatment (6-OHDA_PL; *n* = 10), or (4) a group with a 6-OHDA-induced model of PD and SPD p.o. treatment (6-OHDA_SPD; *n* = 11). A fresh solution of SPD (Sigma-Aldrich, Saint Louis, MO, USA, cat# S0266) in distilled water at a concentration of 10 mg/mL was prepared daily for each rat and administered p.o. at a dosage of 10 mg/kg. Supplementation of SPD or placebo (distilled water) began 24 h after the induction of the PD model and continued for 178 consecutive days. Behavioural tests were conducted 21, 31–33 and 35 days (referred as short-term treatment) and 151, 161–163, and 175 days (referred as long-term treatment) after PD-model induction. Blood was collected from the tail vein (day 38) or heart (day 178), along with brain tissue (day 178), as shown in Figure 7. During the first month after PD-model induction, the rats’ body weights were measured daily and then once a week.

All procedures were approved by the Local Ethical Committee for the Care and Use of Laboratory Animals in Bydgoszcz, Poland 45/2022 and were carried out in accordance with the EU Directive 2010/63/EU. According to this decision, the welfare of the animals was monitored during the experiment. The rats were observed for atypical behaviour (e.g., stereotyped movements, lack of rearing, etc.) and pain daily during recovery from stereotaxic implantation and after returning to home cages during SPD supplementation. Body weight, skin, stool consistency, and urinary signs were analyzed once a week. The endpoints of the procedure were planned if body weight loss exceeded 10% or if the animal exhibited behaviours that impaired its ability to perform essential activities, such as free exploration, grooming, or food and water intake. During both the short and long-term supplementation period, no symptoms were observed that would warrant early termination of the procedure. The animals and collected samples were assigned numerical codes, ensuring that investigators conducting the behavioural, cellular, and biochemical analyses remained blinded to group allocation.

### 4.2. Progressive PD-Model Induction

The rats were anesthetized with 1.5–2.5% isoflurane (Isoflurin, Vetpharma, Barcelona, Spain) (airflow: 0.5 L/min) using an isoflurane vaporizer (Rothacher-Medical, Heitenried, Switzerland) and an oxygen pump (Bitmos OXY 6000, Bitmos GmbH, Düsseldorf, Germany). Analgetic butorphanol at 2.0 mg/kg i.s. (Butomidor, Richter Pharma, Wels, Austria) was administered as described previously [122,123].

The rat was placed in a stereotactic apparatus (Kopf Instruments, Tujunga, CA, USA). The skull was exposed by a midline incision of the skin, and a hole was drilled above the lesion site. The neurotoxin 6-OHDA (6-hydroxydopamine HCl, Sigma–Aldrich, Saint Louis, MO, USA, cat# H4381) was injected into the right dorsal and ventral striatum in a volume of 2 μL in each target (6 μg/μL dissolved in 0.9% NaCl containing 0.02% ascorbic acid), according to the previously described method [124]. The following coordinates from the rat brain stereotactic atlas [125] were used (in reference to the bregma): anteroposterior (AP): +1.6, lateral (L): −2.5, dorsoventral (DV): −4.5 for dorsal and AP: −0.2, L: −3.0, DV: −7.0 for ventral striatum relative to the bregma point. The injections were performed using a microsyringe with a 26-gauge needle (Hamilton Company, Reno, NV, USA) that was attached to a microinjection unit (Model 5000, Kopf Instruments, Tujunga, CA, USA). The injection rate was 0.5 μL/min, and the needle was left in place for an additional 5 min after injection to allow for diffusion into the tissue. To protect the noradrenergic neurons from damage, animals received an intraperitoneal injection with the noradrenaline reuptake inhibitor desipramine (25 mg/kg, Sigma-Aldrich, Saint Louis, MO, USA, cat# D3900) 30 min before neurotoxin injection [126]. The control rats underwent the same procedure but received vehicle (VEH; 0.9% NaCl containing 0.02% ascorbic acid) instead of 6-OHDA. After surgery, the animals were transferred to a warm room, where they stayed until their awakening. The SPD or PL oral supplementation procedures started after a 24 h recovery period from the surgery.

### 4.3. Behavioural Screening for Motor Dysfunction, Anxiety and Anhedonia Level

#### 4.3.1. The Limb-Use Asymmetry Test (Cylinder Test, CT)

The cylinder test device consisted of a transparent plexiglass cylinder with a diameter of 30 cm and a height of 40 cm. For limb use during exploratory activity (touching the wall of the cylinder and landing) and spontaneous rotations, each animal was scored over 5 min. The test was video-recorded using a camera (Canon IXUS 145, Canon, Tokyo, Japan) and manually analyzed by an evaluator blind to the study. The animals were evaluated for asymmetry ratio using the following equation: asymmetry ratio % = [unimpaired − impaired]/both × 100%. Impaired refers to the limb contralateral to the lesioned hemisphere. Both refers to the use of both the impaired and unimpaired limbs during exploratory activity [40].

#### 4.3.2. Elevated Plus Maze (EPM)

The EPM apparatus consisted of two open arms (10 cm in width and 50 cm in length) and two enclosed arms (10 cm in width, 50 cm in length and 40 cm in height), elevated 50 cm above the floor. The EPM was cleaned with 70% ethanol before the start of every trial. The rat was placed in centre square of the maze, always in the same position (heading towards the open end of the maze). Next, the animal was allowed to explore the EPM for 5 min and behaviour was recorded using a video camera (Ikegami, Ikegami Electronics, Neuss, Germany). A video camera was positioned approximately 250 cm over the maze’s centre and connected to a video-tracking digitizing device (EthoVision XT10, Noldus, Wageningen, The Netherlands). The recorded and analyzed reactions included time spent in open/closed arms and the maze’s centre and the number of entries into open/closed arms and the maze’s centre, as previously described [127]. In Section 2 we have presented the number of entries into the open/closed arms of the maze and the time spent in each type of the arms.

#### 4.3.3. Sucrose Preference Test (SPT)

Sucrose consumption is frequently used as indicator of anhedonia in rodents. The SPT was conducted according to the method described by Tadaiesky [128]. During the test, animals had free access to food. Each rat was given two water bottles next to each other during the 24 h training phase to adapt the rats to drinking from two bottles. After training, one of the bottles was randomly changed to one containing a 0.8% sucrose solution and 24 h later the bottles were reversed to avoid the potential preference of drinking liquid from just one bottle. The consumption of water and sucrose solution was estimated simultaneously in each group by daily weighing the bottles. The SPT ratio was defined as follows: sucrose preference percentage (%) = sucrose solution consumption (g)/(sucrose solution consumption [g] + water consumption [g]) × 100%.

### 4.4. Blood and Plasma Collection

Blood samples were collected from the tail vein (day 38) or by heart puncture (day 178) under isoflurane anesthesia (Isoflurin, Vetpharma, Barcelona, Spain) between 08.00 and 10.00 AM. The blood samples were divided into two tubes containing EDTA-K2. One of the tubes was centrifuged (10 min, 3000× *g*) using a Jouan BR4i multifunction centrifuge (Thermo Electron Corporation, Waltham, MA, USA), to obtain fresh plasma without platelets and cells. The supernatant was transferred to Eppendorf tubes, quickly frozen at −70 °C and stored to analyze the plasma cytokines (IL-4 and IL-10) and CORT concentrations. The second part of the sample was tested immediately for peripheral blood morphology and biochemical analysis (whole-blood), and lymphocyte immunophenotyping was carried out by flow cytometry (TCD3^+^, TCD3^+^ CD4^+^ and TCD3^+^ CD8^+^ percentage) in isolated peripheral blood mononuclear cells (PBMCs).

### 4.5. Peripheral Blood Morphology and Biochemistry

An ABX Micros ES 60 (HORIBA Medical, Irvine, CA, USA) hematology analyser was used to determine the count and number of each leukocyte population. The samples were analyzed in duplicate for each rat and at each time point for white blood cell (WBC) number and lymphocyte (LYM), monocyte (MON), and granulocyte (GRAN) count and number determination. The standard markers of liver, kidney, and pancreatic function were measured to evaluate the safety profile of long-term SPD treatment. The whole-blood samples were analyzed using an Exigo C200 (Boule Diagnostics AB, Spånga, Sweden) veterinary clinical chemistry analyser and a comprehensive metabolic panel was carried out, which allows the determination of the following biochemical parameters: ALB—albumin, TP—total protein level, GGT—glutamyltransferase, AST—aspartate aminotransferase, ALT—alanine aminotransferase, ALP—alkaline phosphatase, Crea—creatinine, UA—uric acid, UREA—urea (in the form of urea nitrogen), U/C—urea-to-creatinine ratio, AMY—total amylase, GLU—glucose, TC—total cholesterol, TG—triglycerides, GLOB—globulins, A/G—albumin-to-globulin ratio, TB—total bilirubin.

### 4.6. PBMC Isolation and Flow Cytometry

#### Flow Cytometry Immunophenotyping for TCD3^+^, TCD3^+^ CD4^+^ and TCD3^+^ CD8^+^ Determination

**Figure 8:**
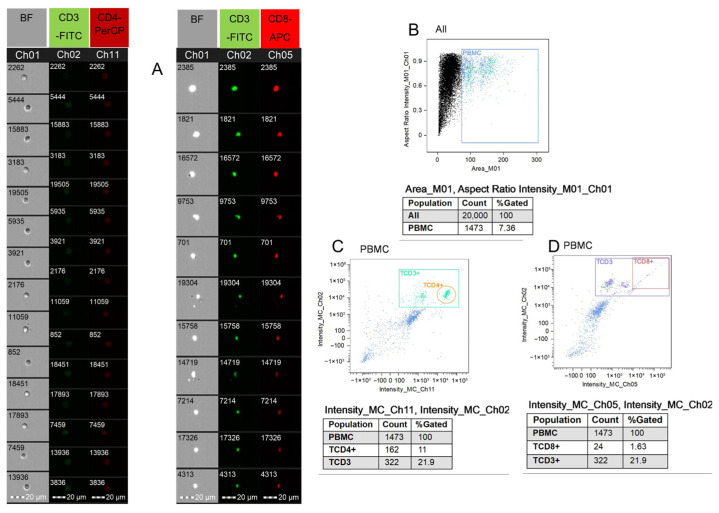
Methods for T cell immunophenotyping using imaging flow cytometry. (**A**) Microphotograph of individual T lymphocytes among CD3^+^CD4^+^ or CD3^+^CD8^+^ populations taken from individual channels at 20× magnification; (**B**,**D**) the gating strategy for CD3^+^CD4^+^ phenotyping; (**B**,**C**) the gating strategy for CD3^+^CD8^+^ phenotyping within the mononuclear lymphocyte population (PBMC). All images taken from Ideas v6.3 software (Amnis, Seattle, WA, USA). Explanation: Ch01, Ch02, Ch05, Ch11—channels used for signal recording.

A volume of 2 mL of peripheral blood was diluted 1:1 in 0.9% NaCl and applied on the Pancoll Rat (PAN-Biotech, Aidenbach, Germany, cat# P04-65500) surface. The PBMCs were separated from blood by the density centrifugation method. After the centrifugation (800× *g*, 30 min at RT), the isolated cells were collected with a Pasteur pipette and washed with BD Pharmingen™ Stain Buffer (BD Biosciences, Franklin Lakes, NJ, USA, cat# 554657) once (350 G, 10 min at RT). A volume of 175 µL of PBMC suspension was labelled with the following antibodies: FITC Mouse Anti-Rat CD3 (BD Biosciences, Franklin Lakes, NJ, USA, cat# 557354), APC Mouse Anti-Rat CD4 (BD Biosciences, Franklin Lakes, NJ, USA, cat# 550057), and PerCP Mouse Anti-Rat CD8a (BD Biosciences, Franklin Lakes, NJ, USA, cat# 558824). All of the antibodies were diluted (1:3) in BD Pharmingen™ Stain Buffer (BD Biosciences, Franklin Lakes, NJ, USA, cat # 554657). The cells were stained for 30 min at RT. The cells were analyzed on the Amnis FlowSight Imaging Flow Cytometer (Luminex, Austin, TX, USA) with four lasers (405 nm, 488 nm, 642 nm and 785 nm). The laser power for the four lasers was 150 mW, 60 mW, 150 mW and 90 mW, respectively. A total of 20,000 events (images; magnification 20×) were acquired from each sample. We used the FlowSight Imaging Flow Cytometer (Amnis, Seattle, WA, USA) with real-time visualization and INSPIRE™ software v. 200.0.336.0 (Amnis, Seattle, WA, USA). The instrument operates as a conventional cytometer but also provides images of every cell tested, acting like a fluorescent and inverted microscope (Figure 8A). Analysis of the T lymphocyte populations was performed using Ideas Application v6.3 (Amnis, Seattle, WA, USA). As a first step, the isolated cells were gated on dot plot Channel 1 (BF) Area (X) and compared to Channel 1 (BF) Aspect Ratio Intensity (Y) to exclude aggregated and damaged cells (Figure 8B). The TCD3^+^CD4^+^ lymphocytes were evaluated on dot plot Channel 11 Intensity (X) (red fluorescence, emission range 642–745 nm), referred to Channel 2 (Y) (green fluorescence, emission range 505–560 nm) (Figure 8C), and the TCD3^+^CD8^+^ lymphocytes were evaluated on dot plot Channel 5 Intensity (X) (red fluorescence, emission range 642–745 nm), referred to Channel 2 (Y) (green fluorescence, emission range 505–560 nm) (Figure 8D). Cells without any staining were used as a negative control and isotype control was applied. Compensation was performed using single-stained samples. The images of the TCD3^+^CD4^+^ and TCD3^+^CD8^+^ lymphocytes and the gating strategy are provided in Figure 8. For statistical analysis, two replications of each sample were used.

### 4.7. Plasma Cytokines and CORT Determination

The concentrations of cytokines and CORT in the plasma were quantified using an enzyme-linked immunoassay method (ELISA) with a commercially available kit for rat IL-4 and IL-10 (Invitrogen, Thermo Fisher Scientific, Waltham, MA, USA, cat# ERA29RB and ERA23RB) and CORT (Cayman Chemical, Ann Arbor, MI, USA, cat# 501320). Samples were prepared according to the manufacturer’s instructions and were analyzed using a Biotek Synergy H1 (Agilent Technologies, Santa Clara, CA, USA) system set to 450 nm (for cytokines) or 412 nm (for CORT) and Gen5 Software (Agilent Technologies, Santa Clara, CA, USA). The cytokines and CORT concentrations were calculated based on the standard curve. The detection sensitivity was 1.5 pg/mL for IL-4, 10 pg/mL for IL-10, and 30 pg/mL for CORT.

### 4.8. Brain Tissue Preparation

The rats were euthanized with Euthasol Vet. (Produlab Pharma B.V., Raamsdonksveer, The Netherlands) at a dose of 120 mg/kg of body weight and perfused transcardially (via the left ventricle) with 200 mL of 0.9% saline, followed by 200 mL of 4% paraformaldehyde in 0.1 M phosphate-buffered saline (PBS Tablets, Merck, Darmstadt, Germany, cat # 524650). The brains were removed quickly, postfixed, cryoprotected in a 30% sucrose solution in PBS, and then frozen and stored at −70 °C until cryostat sectioning (CM 1850, Leica Biosystems, Nussloch, Germany). Coronal 30 µm thick sections containing the SNpc (5.04 mm posterior to the bregma) and CPu (1.20 mm anterior to the bregma) were chosen for TH detection, according to [125].

### 4.9. Immunohistochemistry for TH-Labelled Cells

To determine the loss of dopaminergic neurons in the SNpc and their fibre density (TH-ir) in CPu, we used immunohistochemical staining of the THs previously described [122,129]. Briefly, before all the immunohistochemical stages, the sections were rinsed several times in PBS, then incubated in 0.3% hydrogen peroxide in PBS for 10 min at room temperature and blocked for 45 min with a solution of 5% Bovine Serum Albumin (BSA) (Merck, Darmstadt, Germany, cat# A7030) and 0.3% Triton X-100 (Sigma-Aldrich, Saint Louis, MO, USA) in PBS at room temperature for the effective reduction of non-specific binding. Next, the sections were incubated with a polyclonal rabbit anti-TH antibody (Invitrogen, Thermo Fisher Scientific, Waltham, MA, USA, cat# P21962) at a dilution of 1:1000 (diluted in PBS containing 0.3% TritonX-100 and 3% Goat Serum (Merck, Darmstadt, Germany, G9023) at 4 °C for 2 days. After triple rinsing in PBS, sections were incubated with goat anti-rabbit secondary antibody conjugated with horseradish peroxidase (HRP) (Biorad, Hercules, CA, USA, cat# 1706515, 1:500). The sections were rinsed three times with PBS and incubated in 0.075% diaminobenzidine tetrahydrochloride (DAB) (Merck, Darmstadt, Germany, cat# D5637) in PBS for 5 min. Next, 90 μL of 3% H_2_O_2_ (Eurochem BGD, Tarnów, Poland) was added to 10 mL of the above-mentioned DAB solution to initiate the colour reaction. The reaction was controlled and stopped in PBS buffer when the TH-immunoreactive cells turned brown. The tissue sections were placed on slides, air-dried, and, after dehydration with ethanol, mounted with DPX (DPX new, mountant for histology, Merck, Darmstadt, Germany, cat# 1.00579).

### 4.10. Microscopic Analysis

The labelled TH^+^ cell bodies were analyzed in sections of the SNpc (−5.04 mm from the bregma) and their fibres in the CPu (+1.2 mm relative to the bregma). The photomicrographs of sections were made using a STEMI 508 microscope (Carl Zeiss Microscopy GmbH, Oberkochen, Germany) (magnification 0.5 × 0.65 for CPu and 0.5 × 0.8 for SNpc) with an Axiocam 105 colour camera. The optical densitometry (OD) of TH-ir in the ventral and dorsal striatum was measured using Zeiss Zen 3.5 software (blue edition) in the free version. The grey scale of the TH-positive axonal terminals was performed in the dorsal and ventral striatum every time with the same chosen optical fields of 50.191 μm in both hemispheres. The measured values were corrected for non-specific background staining by subtracting the values obtained from the cortex. The TH OD results are shown as the difference between the signal from the intact side (assumed as 100%) and the signal from the corresponding area in the 6-OHDA- or VEH-treated hemispheres, expressed as a percentage.

### 4.11. Statistical Analysis

The quantitative results are presented as mean values ± standard error (SEM). GraphPad Prism 10.4.1 (Dotmatics, Boston, MA, USA) was used for statistical analysis of the results and their visualization. The normality of the distribution of variables was checked with the Kolmogorov–Smirnov test, and the homogeneity of the variances with Levene’s test. Once both assumptions were met, the analysis was conducted using ANOVA and a post hoc Tukey’s test. Otherwise, the Kruskal–Wallis and post hoc Dunn tests were applied. Statistically significant differences were considered when *p* < 0.05. For both post hoc tests, the *p*-values were calculated in GraphPad Prism with adjustment for multiple comparisons using the Bonferroni correction. Effect sizes were measured using η^2^.

## 5. Conclusions

This study highlights the significant impact of SPD treatment on non-motor functions such as anhedonia and anxiety in the 6-OHDA-injected model of PD. Although the neuroprotective effects of SPD treatment on nigral neurons’ survival during progressive neurodegeneration were not observed, we noted reduced levels of anhedonia and anxiety after long-term SPD treatment in rats. Simultaneous with mood improvement, we demonstrate that SPD reduces peripheral inflammation by influencing the anti-inflammatory properties of lymphocytes and monocytes towards IL-4 and IL-10 systemic secretion. In addition, a reduced level of corticosterone in plasma was observed, which may be responsible for more pronounced exploration in the EPM. Although the mechanism of SPD action may appear more complex, it can affect the supportive functions of TCD4^+^ lymphocytes and monocytes. Pursuing further research to better understand the mechanisms underlying the influence of SPD on various parameters at the systemic level is essential to find a new opportunity for effective supplementation. This could open new therapeutic perspectives for non-motor treatment in PD patients.

## Figures and Tables

**Table 1:** Effects of spermidine treatment on the percentage of the leukocyte population. Granulocyte, lymphocyte and monocyte percentage in the 6-hydroxydopamine (6-OHDA)-injected rats during short- and long-term administration of spermidine (SPD) or placebo (PL) (6-OHDA_PL, *n* = 10; 6-OHDA_SPD, *n* = 11) and in rats from control groups (VEH_PL, *n* = 7; VEH_SPD, *n* = 6). Values are presented as mean ± SEM. Explanations: GRANs—granulocytes, LYMs—lymphocytes, MON—monocytes. ** *p* < 0.01, * *p* < 0.05—difference vs. VEH_PL; ### *p* < 0.001, # *p* < 0.05—difference vs. VEH_SPD; @@@@ *p* < 0.0001, @ *p* < 0.05—difference vs. 6-OHDA_PL, ns—no statistical significance; Dunn post hoc after Kruskal–Wallis test.

Time	Short-Term Administration				
Group	VEH_PL	VEH_SPD	6-OHDA_PL	6-OHDA_SPD	H
GRAN %	0.64 ± 0.04	0.70 ± 0.04	0.65 ± 0.04	0.72 ± 0.04	ns
LYM %	81.03 ± 0.9687	79.92 ± 0.8718	76.63 ± 1.161 *	83.34 ± 0.3627 @@@@ #	H = 33.95; *p* < 0.0001
MON %	0.46 ± 0.02	0.48 ± 0.02	0.46 ± 0.03	0.62 ± 0.04 * @	H = 12.80; *p* < 0.01
**Time**	**Long-Term Administration**				
GRAN %	0.51 ± 0.03	0.39 ± 0.06	0.58 ± 0.05	0.51 ± 0.04	ns
LYM %	79.42 ± 1.331	79.53 ± 0.6047	75.19 ± 0.9402 ** ###	82.05 ± 0.4284 @	H = 21.50; *p* < 0.0001
MON %	1.32 ± 0.45	1.31 ± 0.52	0.79 ± 0.22	1.04 ± 0.36	ns

**Table 2:** Effects of long-term spermidine treatment on biochemical parameter concentrations in peripheral blood. Concentrations of albumin (ALB), alkaline phosphatase (ALP), alanine aminotransferase (ALT), amylase (AMY), aspartate aminotransferase (AST), creatinine (CREA), globulin (GLOB), glucose (GLU), gamma-glutamyl transferase (GGT), total bilirubin (TB), total cholesterol (TC), triglycerides (TG), total protein (TP), uric acid (UA), blood urea nitrogen (UREA), urea/crea ratio (UC), albumin/globulin ratio (A/G) in the 6-hydroxydopamine (6-OHDA)-injected rats during short- and long-term administration of spermidine (SPD) or placebo (PL) (6-OHDA_PL, *n* = 6; 6-OHDA_SPD, *n* = 5) and in rats from control groups (VEH_PL, *n* = 3; VEH_SPD, *n* = 5). Values are presented as mean ± SEM. @ *p* < 0.05—difference vs. 6-OHDA_PL; ### *p* < 0.001, # *p* < 0.05—difference vs. VEH_SPD; ns—no statistical significance; post hoc Dunn following Kruskal–Wallis.

Parameter	Ref	VEH_PL	VEH_SPD	6-OHDA_PL	6-OHDA_SPD	Statistics
ALB g/L	22–48	37.23 ± 1.78	37.32 ± 0.58	37.5 ± 0.4	37.06 ± 0.43	ns
TP g/L	53–69	81.5 ± 5.17	77.38 ± 1.65	74.74 ± 1.34	78 ± 3.29	ns
GLOB g/L	15–42	43.9 ± 4.33	39.65 ± 1.4	37.24 ± 1.39	40.2 ± 2.21	ns
AMY U/L	326–2246	632 ± 55.4	880 ± 104.9	613 ± 34.2	1089 ± 45.0 @	H = 12.12; *p* < 0.01
A/G		0.86 ± 0.06	0.95 ± 0.03	1.02 ± 0.04	0.93 ± 0.03	ns
GGT U/L	0–10	2 ± 0.1	1.85 ± 0.13	2 ± 0	2.67 ± 0.66	ns
AST U/L	39–111	138.33 ± 12.99	145.83 ± 32.11	121.6 ± 19.61	159.66 ± 33.34	ns
ALT U/L	0–80	51.33 ± 5.78	86.33 ± 34.9	40.8 ± 2.46	121 ± 56.07	ns
ALP U/L	<5	6 ± 0.58	5 ± 0.1	6.6 ± 1.6	5 ± 0.1	ns
CREA umol/L	4–57	14.5 ± 2.42	14.35 ± 2.72	18 ± 3.26	16.1 ± 4.17	ns
UA umol/L	48–262	15.33 ± 5.33	25.83 ± 11.11	14.6 ± 3.15	11.66 ± 1.23	ns
TB umol/L	<12	8 ± 0.1	4 ± 1.6	12 ± 0.4 ###	8 ± 0.5	H = 16.08; *p* < 0.01
U/C		349.66 ± 176.47	320.33 ± 102.18	394.8 ± 119.12	433 ± 170.79	ns
GLU mmol/L	2.78–7.50	1.93 ± 0.93	2.52 ± 0.77	2.84 ± 0.32	2.22 ± 0.89	ns
TC mmol/L	0.52–2.38	4 ± 0.4	2.82 ± 0.29	2.34 ± 0.15	2.82 ± 0.41	ns
TG mmol/L	0.30–1.22	0.63 ± 0.33	0.42 ± 0.12	0.38 ± 0.06	0.33 ± 0.02	ns
UREA mmol/L	3.20–8.90	9 ± 0.6	8 ± 0.3	9 ± 0.2	11 ± 0.5 #	H = 8.542; *p* < 0.05

## Data Availability

Raw data are presented as dots in Figure 1, Figure 2, Figure 3, Figure 4, Figure 5 and Figure 6. Additional raw data are available from the corresponding author on reasonable request.

## Abbreviations

The following abbreviations are used in this manuscript:

6-OHDA	6-hydroxydopamine
α-syn	Alpha-synuclein
AAM	Alternatively activated monocytes/macrophages
A/G	Albumin-to-globulin ratio
ALB	Albumin
ALP	Alkaline phosphatase
ALT	Alanine aminotransferase
AMY	Amylase
AP	Anteroposterior
AST	Aspartate aminotransferase
BBB	Blood–brain barrier
BDNF	Brain-derived neurotrophic factor
BSA	Bovine serum albumin
CNS	Central nervous system
CORT	Corticosterone
CPu	Caudate–putamen
Crea	Creatinine
CT	Cylinder test
DAB	Diaminobenzidine tetrahydrochloride
DAT	Dopamine transporter
DSS	Dextran sulfate sodium
DV	Dorsoventral
EAE	Experimental autoimmune encephalomyelitis
ELISA	Enzyme-linked immunoassay method
EPM	Elevated plus maze
FoxP3	Forkhead box protein 3
GGT	Glutamyltransferase
GLOBs	Globulins
GLU	Glucose
GRANs	Granulocytes
H_2_O_2_	Hydrogen peroxide
HPA	Hypothalamic–pituitary–adrenal
HRP	Horseradish peroxidase
IBD	Inflammatory bowel disease
IDO	Indoleamine 2,3-dioxygenase
IL	Interleukin
L	Lateral
LYMs	Lymphocytes
MHC	Major histocompatibility complex
MMP	Mitochondrial membrane potential
MONs	Monocytes
MRSA	Methicylin-resistant *Staphylococcus aureus*
NDs	Neurodegenerative diseases
NF-κB	Nuclear factor kappa-light-chain-enhancer of activated B cells
NMDA	N-methyl D-aspartate acid
NRF2	Nuclear factor erythroid 2-related factor 2
O_2_•^−^	Superoxide
•OH	Hydroxyl radicals
PBMCs	Peripheral blood mononuclear cells
PBS	Phosphate-buffered saline
PD	Parkinson’s disease
ROS	Reactive oxygen species
SEM	Standard error of mean
SNpc	Substantia nigra pars compacta
SPD	Spermidine
SPT	Sucrose preference test
TB	Total bilirubin
TC	Total cholesterol
TGs	Triglycerides
TGF-β	Transforming growth factor beta
Th	T helper cell
TH	Tyrosine hydroxylase
TH-ir	Immunolabeled tyrosine hydroxylase fibre
TH OD	Tyrosine hydroxylase optical densitometry
TNF-α	Tumour necrosis factor alpha
TP	Total protein
Tregs	T regulatory cells
UA	Uric acid
U/C	Urea-to-creatinine ratio
UREA	Urea
UT-B	Urea transporter B
WBCs	White blood cells
